# Enhanced *Bordetella pertussis* acquisition rate in adolescents during the 2012 epidemic in the Netherlands and evidence for prolonged antibody persistence after infection

**DOI:** 10.2807/1560-7917.ES.2017.22.47.17-00011

**Published:** 2017-11-23

**Authors:** Saskia van der Lee, Susanne P. Stoof, Mariette B. van Ravenhorst, Pieter G.M. van Gageldonk, Nicoline A.T. van der Maas, Elisabeth A.M. Sanders, Anne-Marie Buisman, Guy A.M. Berbers

**Affiliations:** 1 Centre for Infectious Disease Control, National institute for Public Health and the Environment (RIVM), Bilthoven, The Netherlands; 2 Department of Peadiatric Immunology and Infectious Diseases, Wilhelmina Children’s Hospital, University Medical Center, Utrecht, The Netherlands

**Keywords:** Pertussis, Epidemiology, Outbreak, Acquisition Rate, Serology, Antibody Decay

## Abstract

In 2012 a large epidemic of pertussis occurred in the Netherlands. We assessed pertussis toxin (PT) antibody levels in longitudinal serum samples from Dutch 10–18 year-olds, encompassing the epidemic, to investigate pertussis infection incidence. **Methods**: Blood was sampled in October 2011 (n = 239 adolescents), then 1 year (2012; n = 228) and 3 years (2014; n = 167) later. PT-IgG concentrations were measured by immunoassay and concentrations ≥50 IU/mL (seropositive) assumed indicative of an infection within the preceding year. **Results**: During the 2012 epidemic, 10% of participants became seropositive, while this was just 3% after the epidemic. The pertussis acquisition rate proved to be sixfold higher during the epidemic (97 per 1,000 person-years) compared with 2012–2014 (16 per 1,000 person-years). In 2012, pertussis notifications among adolescents nationwide were 228/100,000 (0.23%), which is at least 40 times lower than the seropositivity percentage. Remarkably, 17 of the 22 seropositive participants in 2011, were still seropositive in 2012 and nine remained seropositive for at least 3 years. **Discussion**: Longitudinal studies allow a better estimation of pertussis infections in the population. A PT-IgG concentration ≥50 IU/mL as indication of recent infection may overestimate these numbers in cross-sectional serosurveillance and should be used carefully.

## Introduction

Pertussis, caused by the bacterium *Bordetella pertussis*, is a vaccine preventable infection of the upper respiratory tract, which is particularly severe in young infants [1,2]. Despite high vaccination coverage, pertussis has re-emerged since the 1990s in most industrial countries [3-7].

Serological surveillance studies are a tool to investigate the pertussis infection rate in the population by determining serum IgG antibody concentrations against pertussis toxin (PT). PT is one of the major virulence factors during infection and is only expressed by *B. pertussis* [8-11]. A cross-sectional population-based serosurveillance study conducted in the Netherlands in 2006–2007, showed that 9% of adolescents (10–18 years of age) and adults (> 18 years of age) contained a PT serum antibody concentration ≥ 50 IU/mL, suggestive for a pertussis infection in the preceding year [12]. In contrast, the number of reported pertussis cases during that same period was only 0.03% (30 cases/100,000 persons) [13]. More accurate information regarding pertussis acquisition can be provided by longitudinal studies. Furthermore, longitudinal studies may also illustrate the course of antibody kinetics during pertussis infections and between epidemics.

In 2011–2012, a longitudinal meningococcal vaccination trial was conducted among Dutch adolescents [14]. By coincidence, two blood samples from that study were collected encompassing the largest pertussis epidemic in the Netherlands since its resurgence. During that 2012 epidemic, the number of reported pertussis cases increased to 83/100,000 persons in the whole Dutch population [13]. In addition to the samples from 2011 and 2012, a follow-up sample was collected in 2014, resulting in three longitudinal samples over a period of three years. We determined PT specific IgG antibody concentrations in these samples to investigate the (sub-clinical) pertussis infection rate among adolescents during and after the 2012 epidemic and to explore antibody kinetics after pertussis infection.

## Methods

### Study design and participants

The samples used in this study originated from a phase IV meningococcal serogroup C conjugated (MenCC) booster vaccine trial [14,15]. In short, adolescents aged 10, 12 and 15 years were vaccinated in October 2011 with a MenCC booster vaccination and blood samples were collected before, 1 month, 1 year and 3 years following vaccination. Participants were randomly selected from four different municipalities in the Netherlands. Samples collected one month post-booster vaccination were excluded in the current study. Time of sampling is further indicated by year of sampling.

The study was approved by the Medical research Ethics Committees United (MEC-U, Nieuwegein, the Netherlands). Written informed consent was obtained from both parents and from participants aged 12 years and older. The trial was registered at the European Clinical Trials Database (2011–000375–13) and at the Dutch Trial Register (www.trialregister.nl; NTR3521).

### Vaccination background

All participants were vaccinated according to the Dutch national immunisation program (NIP), including four vaccinations with the whole-cell pertussis combination vaccine (DTwP-IPV-Hib, the Netherlands Vaccine Institute (NVI), Bilthoven, the Netherlands) in a 3 + 1 schedule in the first year of life. In addition, the 10 and 12 year-olds received an acellular pertussis vaccine in combination with a DT-IPV booster (NVI) at 4 years of age.

### Pertussis toxin specific antibody concentration

Serum PT-IgG antibody concentrations were measured using the fluorescent-bead-based multiplex immunoassay as described [16]. The in-house pertussis reference sample was previously calibrated to United States reference pertussis antiserum human lot 3 (Center for Biologics Evaluation and Research, Food and Drug Administration, Silver Spring, Maryland, United States). To express PT-IgG concentration in international units (IU) per mL, we extensively compared the in-house reference to the World Health Organization international standard (pertussis antiserum 1^st^ international standard, 06/140, National Institute for Biological Standards and Control, Potters Bar, United Kingdom [17]). We found a small difference with the previous calibration resulting in a correction of a factor 0.8 for PT antibody levels. This implies that the level for recent *B. pertussis* infection changes from 62.5 enzyme-linked immunosorbent assay units (EU) per mL to 50 IU/mL in our laboratory [12,18]. A PT-IgG concentration of ≥ 50 IU/mL was used as a cut-off for pertussis infection in the preceding year [12] with a specificity of 95% and a sensitivity of 80%, and indicated here as seropositive. Furthermore, a PT-IgG concentration of ≥ 100 IU/mL was used as cut-off for pertussis infection in the preceding 6 months [12], with a specificity of 99% and sensitivity of 70% [19].

### Pertussis surveillance data

To compare the number of seropositive study participants with the reported notifications of all Dutch adolescents of 10–18 years of age, pertussis notification data were extracted from the mandatory national surveillance notification system for vaccine-preventable diseases in the Netherlands, as previously described [7]. Then, based on the age of the study participants, all cases who matched in age were grouped per year. In this study, the provided pertussis incidence data were obtained from November to October, as the serology data were also determined in the month October. For example, incidence data of all Dutch 11, 13 and 16 year-olds, which were reported between November 2011 and October 2012, were used for the comparison with serology data at sampling time point October 2012, while incidence data of all Dutch 13, 15 and 18 year-olds reported between November 2013 and October 2014 were used to compare with the serology data at sampling time point October 2014.

### Statistical analyses

Differences in PT-IgG antibody concentrations between the age groups were tested with one-way analysis of variance (ANOVA). PT-IgG antibody concentrations were also dichotomised to study differences in proportion of participants with a PT-IgG concentration ≥ 50 IU/mL or with a PT-IgG ≥ 100 IU/mL between time points, and were tested using McNemar tests. A p-value below 0.05 was considered statistically significant. Chi-squared tests were used to assess difference according to sex within the study population.

The participants who became seropositive during the course of the study were considered to have been infected with *B. pertussis*. The acquisition rates per 1,000 person-years between October 2011 and October 2012 and between October 2012 and October 2014 were determined by dividing the number of new pertussis infected individuals by the total person-time in years of all initially negative individuals [20].

## Results

### Study population

Of the 268 participants enrolled in the original study, 29 had not given permission for sample analysis beyond the objectives for the meningococcal vaccine trial and were therefore excluded from analysis [14]. From the 239 participants available for analysis, blood samples had been collected in 2011 (n = 239), 2012 (n = 228; 95.4%) and 2014 (n = 167; 69.9%). In total 72 participants were lost to follow-up during the course of the study. These were distributed evenly across all age groups. Baseline characteristics are listed in Table 1. There were no differences according to sex within the overall study population (p = 0.134). At the beginning of the study (2011), the PT-IgG geometric mean concentrations (GMCs) were similar between the age groups (GMC of the 10, 12 and 15 year-olds was 5.4, 6.8, and 6.7 IU/mL respectively, p = 0.420), and between males and females (GMCs were 5.8 and 6.7 IU/mL, respectively, p = 0.276).

**Table 1:** Characteristics of participants at the beginning of the study, the Netherlands, October 2011 (n = 239 participants)

Characteristics	Overall	10 year-olds	12 year-olds	15 year-olds
Number of participants^a^	239	81	82	76
Mean age in years in October 2011 (SD)	NA	9.9 (0.3)	12.0 (0.3)	15.0 (0.3)
Number of participants of male sex (%)	120 (50)	34 (42)	42 (51)	44 (58)

NA: not applicable; SD: standard deviation.

^a^ All participants were primed with a whole-cell pertussis combination vaccine four times in the first year of life (3 + 1 schedule). Ten and 12 year-old participants received an acellular pertussis booster vaccine at 4 years of age.

### Pertussis surveillance data

**Figure 1:**
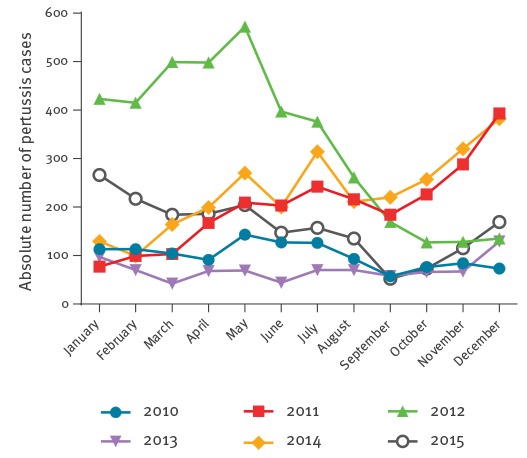
Absolute number of reported pertussis cases for Dutch adolescents 10 to 18 years of age, January 2010–December 2015 (n = 13,127 total cases)

The number of notified pertussis cases in Dutch adolescents aged 10–18 years for the period 2010–2015 is depicted in Figure 1. During the 2012 epidemic, a twofold increase in the number of reported pertussis cases was seen in the respective age range (10–18 years of age) of the study participants, with 115/100,000 (0.12%) cases between November 2010 and October 2011, and 228/100,000 cases (0.23%) between November 2011 and October 2012. After the epidemic, between November 2013 and October 2014, the number of reported cases was 80/100,000 (0.08%).

### Pertussis toxin IgG seropositivity per year

In October 2011, 9% (22/239) of the participants had a PT-IgG concentration ≥ 50 IU/mL. The proportion of participants with a PT-IgG concentration ≥ 50 IU/mL in 2012 was significantly higher compared with 2011 (17%, 39/228; p = 0.002). In 2014, 13% (21/167) of the participants had a PT-IgG concentration ≥ 50 IU/mL (p = 0.238 and p = 0.167 compared with 2011 and 2012 respectively). The proportion of participants with a PT-IgG concentration ≥ 100 IU/mL was highest in 2012 (12%, 27/228) compared with 2011 and 2014 (5% (13/239), p = 0.004 and 5% (8/167), p = 0.017, respectively). For both cut-off PT-IgG concentrations, no differences in seropositivity were found according to sex.

### New pertussis infections and acquisition

**Figure 2:**
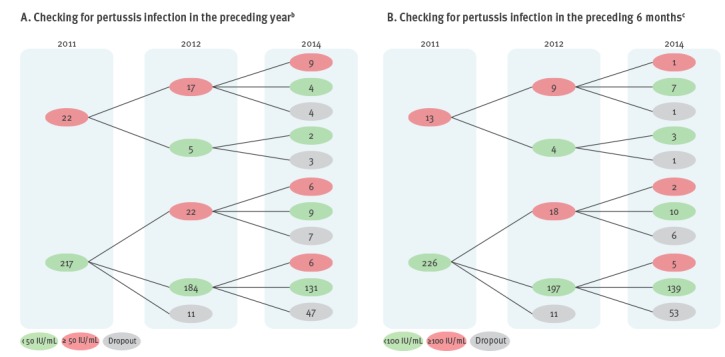
Follow up at different time points of Dutch study participants aged between 10 and 18 years for pertussis infection, the Netherlands, 2011–2014 (n = 239 initial participants^a^)

Of the initially negative participants in 2011, 10% (22/217) were seropositive in 2012, resulting in an acquisition rate of 97 per 1,000 person-years (Figure 2a). Of the initially negative participants in 2012, 3% (6/184) became seropositive in 2014 (Figure 2a). The acquisition rate for the period 2012–2014 was 16 per 1,000 person-years. During the 2012 epidemic, the percentage of newly seropositive participants was 44 times higher than the percentage of reported pertussis cases in these age groups.

The red circles indicate the number of participants above the cut-off; the green circles the number of participants below the cut-off. The number of participants lost to follow up (dropout) are depicted in grey.

^a^ A total of 72 participants were lost to follow-up during the course of the study.

^b^ Pertussis toxin (PT)-IgG concentration ≥ 50 IU/mL were taken as indicative of a pertussis infection in the preceding year.

^c^ PT-IgG concentration ≥ 100 IU/mL were taken as indicative of a pertussis infection in the preceding six months.

### Kinetics of pertussis toxin specific antibody concentrations

**Figure 3:**
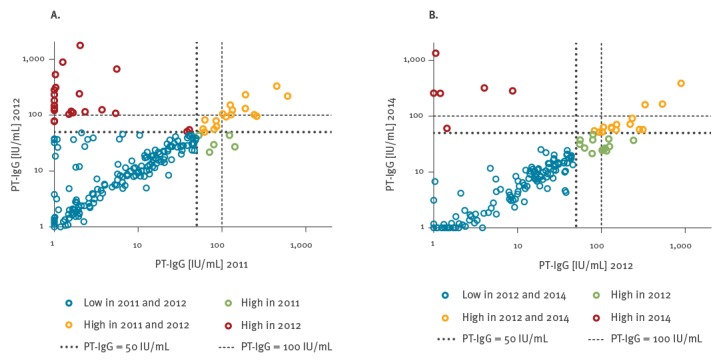
Individuals’ paired pertussis toxin (PT) specific IgG concentrations (IU/mL) in A) 2011 vs 2012, and B) 2012 vs 2014, the Netherlands, 2011–2014 (n = 239 initial participants^a^)

Of the 22 participants who became seropositive (≥ 50 IU/mL) in 2012, 20 had a PT-IgG concentration < 10 IU/mL in 2011. Eighteen of these 22 participants had a PT-IgG concentration > 100 IU/mL in 2012 (red dots Figure 3a). All participants who became seropositive between 2012 and 2014 (n = 6) had PT-IgG concentrations < 10 IU/mL in 2012 (red dots in Figure 3b).

IU: international units.

‘High’ is ≥ 50 IU/mL, ‘low’ is < 50 IU/mL.

^a^ A total of 72 participants were lost to follow-up during the course of the study.

PT-IgG levels in the samples of the 22 seropositive participants in 2011 declined on average 1.8 fold between 2011 and 2012 (orange and green dots in Figure 3a), and five samples dropped below 50 IU/mL in 2012 (green dots in Figure 3a). Three seropositive participants in 2011 showed a 1.2–1.3 fold increase in PT-IgG concentrations in 2012. An average of 3.0 fold antibody decay was observed between 2012 and 2014 in the 39 seropositive participants of 2012 (orange and green dots in Figure 3b).

### Duration of seropositivity

In 2012, 17 of the 22 participants with a PT-IgG concentration ≥ 50 IU/mL in 2011 maintained a PT-IgG concentration ≥ 50 IU/mL (Figure 2a). Of the 39 participants with a PT-IgG concentration ≥ 50 IU/mL in 2012, 15 maintained this PT-IgG concentration ≥ 50 IU/mL in 2014. Furthermore, nine of the 22 participants with a PT-IgG concentration ≥ 50 IU/mL in 2011 maintained a PT-IgG concentration ≥ 50 IU/mL over the 3-year course of the study (Figure 2a). One participant (1/13) maintained a PT-IgG concentration ≥ 100 IU/mL for 3 years (Figure 2b). In 2012, nine of the 13 participants in 2011 maintained a PT-IgG concentration ≥ 100 IU/mL and three of the 27 participants in 2012 maintained a PT-IgG concentration ≥ 100 IU/mL in 2014 (Figure 2b).

## Discussion

In this study, PT specific antibody levels in longitudinal samples from adolescents aged 10–18 years encompassing the pertussis epidemic of 2012 in the Netherlands were assessed. The pertussis infection acquisition rate in the study population was six times higher during the 2012 epidemic (97 per 1,000 person-years), compared with the low-epidemic period of 2013–2014 (16 per 1,000 person-years). Interestingly, 17 of the 22 seropositive participants in 2011 were still seropositive in 2012 and nine remained seropositive for three years.

In cross-sectional serosurveillance studies a PT-IgG concentration of ≥ 50 IU/mL is defined as cut-off for *B. pertussis* infection in the preceding year [11]. By applying this cut-off to our cohort, even in low-epidemic years (2011 and 2014) compared with 2012, already 9 (22/239) to 13% (21/167) of the study participants could be considered to be recently infected with *B. pertussis*. This is in line with the cross-sectional serosurveillance study conducted in 2006–2007 in the Netherlands [12] and with other studies from Australia (2007) [21], Belgium (2012) [22], and Norway (2004) [23]. At the end of the pertussis epidemic in 2012, the proportion of seropositive adolescents in our cohort almost doubled to 17% (39/228). Moreover, 10% (22/217) of the participants had become seropositive in 2012. This indicates that these participants were actually infected with *B. pertussis* during the epidemic and that pertussis circulation was high. In contrast, only 3% (6/184) of the participants became seropositive between 2012 and 2014. Acquisition rates were determined based on initial seronegative participants, which included the participants who were unavailable for additional blood samplings during the rest of the study period. Approximately 30% (72/239) of the recruited participants were lost to follow-up between 2011 and 2014. The percentage of participants who became seropositive during the study could possibly have been even higher than indicated, and thereby also the pertussis acquisition rate, as we do not know the status of these participants. 

The high pertussis acquisition rate in adolescents observed during the epidemic could be caused by the relatively easy spreading of *B. pertussis* via respiratory droplets [24] and the tendency of adolescents to mix especially with people of the same age [25]. However, in a cross-sectional population-based serosurveillance study no differences were found in the percentage of seropositive participants between adolescents, adults and elderly [12]. This suggests that pertussis acquisition might be comparable for all individuals above 9 years of age due to high transmission through all kinds of routes.

Following individual antibody concentrations, we noticed limited antibody decay in seropositive samples during the three year follow-up. Nine of the 22 initially seropositive participants in 2011 remained seropositive for at least three years. This was in agreement with a previous study, where the half-life of specific PT-IgG antibodies after infection was estimated to be ca 17 months [26]. Although natural boosting of these nine participants during the study period cannot be excluded, particularly during the epidemic, no increase in PT-IgG antibody concentration was found between 2012 and 2014. This finding emphasises that the use of a PT-IgG concentration of ≥ 50 IU/mL as a cut-off for pertussis infection in the preceding year leads to an overestimation of pertussis infections. Moreover, a PT-IgG antibody concentration above 100 IU/mL, used as indication of pertussis infection in the preceding six months [11,12], will also result in an overestimation of pertussis infections. At this moment, it seems unclear which PT-IgG cut-off is appropriate to use as an indication for recent pertussis infection.

Since the scope of the original study was to investigate immune responses to a meningococcal serogroup C booster vaccination, information about clinical manifestation and PCR confirmation of pertussis was unavailable. Therefore, we do not know from the seropositive participants if the pertussis infection involved symptoms or not and if they were able to transmit *B. pertussis* to others. Presumably most of the infected participants were asymptomatic or only had mild symptoms, as the majority of pertussis infections do not cause severe morbidity in adolescents [27]. It should be noted that pertussis immunisation schedules differed among the 10, 12 and 15 year-old age groups in this study. In 2001, a booster dose with the acellular pertussis vaccine at the age of four years was introduced into the Dutch NIP. Participants aged 15 years at the beginning of the study, were five years of age in 2001 and therefore did not receive this booster. However, PT specific antibodies wane rapidly after acellular pertussis booster vaccination [28,29], and the booster vaccination has a limited duration of protection [30,31]. Moreover, no differences were found in PT-IgG concentrations between the three age groups at the beginning of the study (2011). Therefore, the effect of this acellular pertussis booster vaccination on the pertussis susceptibility of the participants in our study is likely limited.

Although our findings suggest that numbers of recent pertussis infections indicated in cross-sectional serosurveillance studies could be overestimated, we have provided evidence that the pertussis acquisition rate was 97 per 1,000 person/years during the 2012 pertussis epidemic in our participants, over 40 times higher than the actually reported pertussis cases in these age groups. This suggests that also adolescents could form a large reservoir for *B. pertussis*, which poses a possible threat for young unvaccinated infants who are especially at risk of developing severe illness [32]. In order to reduce this risk in neonates, several countries have implemented adolescent pertussis booster vaccinations next to maternal vaccination [33,34]. Unfortunately, whether (repeated) administrations of acellular pertussis booster vaccines can reduce the circulation of *B. pertussis* remains uncertain. In a baboon model, Warfel et al. demonstrated that acellular pertussis vaccines protected against disease, but did not stop transmission and colonisation, while whole-cell pertussis vaccines protected against disease with rapid clearance of *B. pertussis* [35]. Furthermore, individuals primed only with acellular pertussis vaccines in the first year of life have an increased risk of acquiring pertussis compared with individuals vaccinated with at least one whole-cell pertussis vaccine [36-38]. Nowadays, industrialised countries use acellular pertussis vaccines in the first year of life for priming. Therefore, enhanced surveillance of pertussis acquisition rates is crucial to monitor pertussis circulation in a population increasingly immunised with the acellular pertussis vaccine.

In conclusion, our results demonstrate that using longitudinal serological studies the acquisition rate of (sub-clinical) pertussis infections can be determined. Thereby, these studies can contribute to a better estimation of the true pertussis incidence in the population. Pertussis incidence in adolescents proved much higher than the number of reported pertussis cases, especially during an epidemic. This indicates that protection against infection conferred by the Dutch national immunisation programme is limited at that age. Furthermore, we highlighted to be cautious applying the current PT-IgG cut-off values in serosurveillance studies, as this will result in an overestimation of the numbers of pertussis infections.
